# Supplementary material for: Distress Levels of Parents of Children with Neurodevelopmental Disorders during the COVID-19 Pandemic: A Comparison between Italy and Australia
Source: Int J Environ Res Public Health. 2021 Oct 21;18(21):11066. doi: 10.3390/ijerph182111066 (PMC8583226; doi:10.3390/ijerph182111066)
Supplement: Supplementary file 1 [file ijerph-18-11066-s001.zip › ijerph-1378448-supplementary.pdf]

**Supplementary Materials**

**Table S1.** Impacts on children and families (those who agree or strongly agree with each statement)

| | Italy | | Australia | |
|---|---|---|---|---|
| | n | % | n | % |
| **General** | | | | |
| My child’s overall health and wellbeing has been impacted by the COVID-19 crisis | 117 | 58.5 | 200 | 69.4 |
| COVID has worsened pre-existing health conditions for my child | 91 | 45.5 | 139 | 50 |
| COVID has had an impact on my wellbeing | 95 | 47.7 | 197 | 68.4 |
| COVID has had an impact on my other children’s wellbeing | 56 | 36.4 | 164 | 57.1 |
| **Types of support** | | | | |
| My support networks have decreased significantly due to COVID | 97 | 53.0 | 178 | 77.4 |
| COVID has disrupted carer supports and services | 79 | 44.9 | 127 | 71.8 |
| COVID has disrupted respite arrangements | 52 | 27.1 | 91 | 72.2 |
| COVID has significantly disrupted the allied health services my child accesses (e.g. occupational therapy, speech therapy) | 78 | 74.3 | 151 | 72.2 |
| COVID has significantly disrupted my child’s psychological therapy | 42 | 56.0 | 114 | 68.7 |
| COVID has significantly disrupted my child’s behavioural therapy | 58 | 70.7 | 97 | 74.1 |
| My child has started taking more medication than normal due to the COVID pandemic | 26 | 25.2 | 232 | 97.1 |

| | | | | |
|---|---|---|---|---|
| My child is taking less medication than usual because of the COVID pandemic | 3 | 3.1 | 236 | 98.7 |
| My child's ability to access specialists has been significantly impacted due to COVID | 52 | 29.2 | 148 | 70.5 |
| My ability to organize supports has been significantly impacted due to COVID (e.g. support workers) | 41 | 28.1 | 114 | 71.7 |
| COVID has significantly disrupted my child's other treatments and supports (e.g. physiotherapy, OT, speech therapy) | 60 | 64.5 | 150 | 81.5 |
| My child's contacts with people outside of the home have reduced since before the COVID pandemic | 117 | 62.2 | 228 | 95.8 |
| **Family health** | | | | |
| COVID has significantly disrupted my child's routines | 138 | 69.0 | 243 | 94.9 |
| During the past two weeks, COVID restrictions have been stressful for my child | 93 | 47.0 | 191 | 75.5 |
| Home isolation has been stressful for me | 119 | 60.1 | 183 | 72.335 |
| Balancing work with childcare/family responsibilities has been difficult for me due to COVID | 74 | 40.0 | 186 | 80.9 |
| My pre-existing mental health conditions have been worsened due to COVID | 38 | 32.5 | 110 | 57.0 |
| My pre-existing physical health conditions have been worsened due to COVID | 34 | 28.6 | 90 | 46.6 |
| My family is feeling worried about COVID | 122 | 61.6 | 146 | 57.9 |
| The COVID restrictions have had significant negative effect on my life | 114 | 57.6 | 130 | 51.8 |
| The amount of quality time I spend with my child has reduced since before COVID-19 | 14 | 7.1 | 47 | 19.0 |

| | | | | |
|---|---|---|---|---|
| Changes related to the Coronavirus/COVID-19 crisis have created financial problems for me or my family | 65 | 32.8 | 104 | 43.5 |
| The COVID situation has resulted in changes to the employment status of people in my household | 96 | 48.5 | 100 | 48.8 |
| I am concerned about the stability of our living situation | 52 | 26.3 | 72 | 31.3 |
| I am worried about running out of food due to a lack of money | 21 | 10.6 | 34 | 14.7 |
| I am hopeful that the COVID crisis will end soon | 189 | 94.5 | 186 | 73.2 |
| I am worried about making it through future periods of isolation | 122 | 61.9 | 122 | 48.2 |
| **Home based learning** | | | | |
| COVID has prevented my child from attending educational facilities in person (e.g. childcare, school) | 176 | 90.3 | 224 | 91.1 |
| COVID has disrupted my child's learning | 135 | 68.9 | 212 | 83.8 |
| My child has completed home-based learning during the COVID-19 pandemic. | 170 | 87.2 | | |
| I have adequate access to internet and online resources | 163 | 84.0 | 217 | 87.2 |
| My child has adequate capacity to engage in home-based learning | 93 | 47.5 | 110 | 44.4 |
| I have adequate capacity to support my child's educational needs | 155 | 79.9 | 117 | 46.4 |
| **Child behaviours** | | | | |
| My child has experienced reductions in sleep quality as a result of COVID-19 | 37 | 18.7 | 114 | 46.9 |
| My child's exercise has decreased as a result of COVID-19 | 135 | 67.5 | 169 | 68.7 |

| | | | | |
|---|---|---|---|---|
| My child's diet has been poorer as a result of COVID-19 | 22 | 11.2 | 82 | 33.7 |
| My child's ability to spend time outside has reduced as a result of COVID. | 161 | 80.9 | 138 | 55.9 |
| My child has difficulty following the recommendations for keeping away from close contact with people | 52 | 27.5 | 136 | 57.1 |
| My child has had difficulty maintaining relationships as a result of COVID | 83 | 43.2 | 163 | 67.9 |
| My child has become easily annoyed, irritable, and angry since the COVID-19 outbreak, compared to before the outbreak | 120 | 60.6 | 159 | 65.2 |
| The cancellation of important events (such as holiday, birthday celebration etc.) has been difficult for my family | 52 | 28.0 | 159 | 63.1 |
| My child has been watching significantly more TV or digital media (e.g., Netflix, YouTube, web surfing) since before COVID | 144 | 73.1 | 203 | 82.2 |
| My child has been using significantly more social media (e.g., Facetime, Facebook, Instagram, Snapchat, Twitter, TikTok) since before COVID | 63 | 43.5 | 74 | 45.7 |
| My child has been playing significantly more video games since before COVID | 101 | 58.7 | 132 | 65.4 |
